# A waiting crisis?

**DOI:** 10.1016/S0140-6736(23)00238-6

**Published:** 2023-02

**Authors:** Laura Salisbury, Lisa Baraitser, Jocelyn Catty, Kelechi Anucha, Stephanie Davies, Michael J Flexer, Martin D Moore, Jordan Osserman

**Affiliations:** Department of English and Creative Writing (LS), Wellcome Centre for Cultures and Environments of Health (LS, KA, MJF, MDM), University of Exeter, Exeter EX4 4QH, UK; Department of Psychosocial Studies, Birkbeck, University of London, London, UK (LB, SD, JO); Tavistock and Portman NHS Foundation Trust, London, UK (JC)

It is a repeated refrain across current UK news: waiting times for health care in the National Health Service (NHS) have reached crisis levels. Millions of patients are waiting for elective surgery and potentially hundreds of people could be dying each week due to excessive waits for ambulances and emergency care. Concerns about waiting lists and waiting times are hardly new, particularly in systems that offer universal access to health care funded by general taxation. In lower-income countries or those that fund health care through private health insurance, concerns about gaps in care delivery may be less focused on waiting than on how some needs are fundamentally excluded from the system. But as the NHS’s promise of universal care is challenged by insufficient health and social care budgets, long-term underinvestment in the face of the complex needs of an ageing population and rising demand for services, severe staff shortages, and the worsening health of those most affected by widening inequalities, the UK finds itself, as it looks to recover from the COVID-19 pandemic, in what is being characterised as the biggest crisis of waiting since the inception of the NHS.

No-one should wait in situations that are medically dangerous, in intolerable pain, or when a quick intervention will improve clinical outcomes. Being made to wait can be neglectful of legitimate patient needs. But although waiting is often used to mark the success or failure of care in the NHS, waiting is not simply care’s opposite. Instead, as all health-care practitioners know, waiting is intrinsic to care. It is there in the extended time needed for therapy or therapeutics to work; in the watchful waiting before or after diagnosis; and in the time that stretches through remission, relapse, or palliative care. Even within the most urgent medical interventions, waiting is crucial. Cardiopulmonary resuscitation during cardiac arrest relies on a tiny hiatus between chest compressions mimicking the interval between heart-beats: a wait that can feel like a lifetime. At the other end of the spectrum, many patients in general practice have complex, chronic conditions that are often resistant to straightforward interventions, but instead require long-term treatment, monitoring, and management. Here, clinicians’ capacity to wait with, or sometimes simply to continue to be available to their patients, is the treatment on offer. In child and adolescent mental health services in the UK, where referrals have increased significantly, clinicians endeavour to find ways to mitigate crisis for those with serious mental health needs while managing parents’ anxieties in the hope of preventing emergency presentations. But, even when it comes, adolescent mental health care does not usually involve a quick fix; instead, interventions take place over months and years, opening up time for thought and development so that envisaging the future becomes possible again.

Waiting Times—a Wellcome Trust-funded research project on the relationship between time and health care—has found that, for both clinicians and patients, practices of pausing, delaying, slowing, and stretching time are vital to care. Although it is crucial that patients have access to high-quality care when needed, we argue that understanding how waiting is intrinsic to care, rather than “dead” time, enables a deeper understanding of what timely care might be—not only for patients, but also for the NHS and its social mission.

Even at the inception of the NHS, there was a concern that universal care would lead to excessive waiting; but new ways of framing the experience also emerged. As the NHS attempted to repair the damage to the nation’s health produced by centuries of unequal access to treatment, it was acknowledged that waiting was sometimes the cost of universal care. Waiting seemed more tolerable in the knowledge that you were now a patient of the NHS, whose needs had been assessed, rather than someone who might never afford health care. Waiting had a place as a collective practice within a shared, social project. By the 1980s, however, with new managerial cultures and political imperatives, waiting overwhelmingly came to signal service failure. This idea emerged against a backdrop of accelerated social change and technologically driven increases in the pace of life that made waiting ever harder to bear.

Much philosophical and social science research tells us that the tolerability of waiting is determined not so much by the quantity of time one is asked to endure, but its quality. Subjectively, pain can slow time; waiting also becomes unbearable when what you are waiting for seems unlikely to be delivered in a timely way. Yet modes of waiting with others that involve paying careful attention to making relationships can manage, ameliorate, and make therapeutic use of time. These relational practices of making time are there in the everyday work of returning and checking to understand what is needed at any given moment; they are there, too, in the personal letter from a clinic that reminds someone on a list that they are indeed someone’s patient and being kept in mind. Time may not flow smoothly and may even feel stuck in such moments. But for the young person who is not sure that life is worth living but turns, time and again, to their psychotherapist or mental health team; for the adult telling their doctor about a series of chronic conditions who comes to learn that their experience will be heard and attended to; or for the person in a hospice sharing their life story with an attentive carer: in these moments of relating, time is created rather than wasted. The practices of waiting that nurture such relationships are the ground on which care grows.

So how do we navigate the idea that waiting lists and times are crises that demand urgent forms of action, while also taking care of the waiting intrinsic to care? The crisis account feels both accurate and timely as we look towards a post-pandemic future, but its call for immediate action also has risks. These include opening the door to further privatisation of the NHS to restructure the system, the hardening of criteria around who is eligible for care, and more unsustainable sacrifices being demanded of its understaffed and undervalued workforce. At such a moment, in addition to the need for long-term investment in the NHS and social care, preventive approaches, and improved public health, we suggest that it is vital to develop more nuanced narratives about crisis, waiting, and care. Only then will we be able to preserve the crucial difference between waiting that can and should be ameliorated by improving resourcing and systems, and waiting that is central to individual and collective care.

The word crisis comes from the Greek *krinein* (to decide, distinguish, separate). In its original Hippocratic framing, crisis was a moment of medical diagnosis or judgement: a turning-point when life and death hung in the balance. The concept was taken up politically during the Enlightenment, when not just the human body but history itself became something we could diagnose and act upon. But there is something intrinsic to the structure of crisis that makes a crisis-free future hard to produce. If crisis framings circumscribe the terms of the problem, prescribing which actions are legitimate to address it, only certain responses will be deemed appropriate to its urgency. There is thus a swing between crisis-diagnosis and crisis-action to address or manage it. Crisis produces more crisis.

In the UK context, waiting has historically held a specific value in health-care practices and institutions built on Christian ideas and traditions of care and charity. Being a “patient” meant enduring within a broader framework that emphasised the possibility and expectation of deliverance. But as waiting for a Christian God lost hold in many western cultures in the late 19th and 20th centuries, waiting became more associated with the redundancies built into standardised or industrialised time regimes. Waiting was imagined as “dead time”—the opposite of action, agency, and progress. Today, as narratives of time circulate between the traumas of the past, the urgencies of the present, and anxieties about a foreclosed future, time is often experienced as paradoxically both speeded up and stuck. With the loss of overarching religious frameworks and ideas of historical progress, it can feel as if there is no time to wait, but waiting is all we can do.

Obvious crisis responses to the NHS waiting crisis are to reduce it via restructuring, reallocating, or rationing, as time becomes a finite resource that must be spent efficiently to move patients via pathways through the system. But moving people through the system faster does not address the problem of care at the root of the waiting crisis. Care is not simply linked to a quantity of time; instead, care depends on a quality of time made between people that needs expansion and deepening rather than contraction. The relational qualities of care and time matter. To produce a future that moves beyond crisis, we need to think beyond the economics of time as a finite resource towards understanding how time is produced in and through care.

Health-care practitioners know much about making and remaking relationships in the slow, repetitive time of staying alongside the chronically unwell, those at the end of life, and those who feel that life is no longer worth living. Similarly, those in the NHS know much about the day-to-day experiences of having to endure what feels like wasted time, as the service juggles needs, insufficient resources, and commitments to a universal service. One might say that there is a particular “NHS-ness” to these experiences, as both individual health improvement and service change often feel almost imperceptible and require patience, from both practitioners and patients. Because neither waiting nor health care is equitably spread in the NHS, care may also have to be carved out in what feels like the margins of the service. Some practitioners and patients find themselves offering and accessing care while working around systemic racism that leaves racially marginalised people experiencing treatments and interactions that feel neither timely nor caring. The NHS-ness of waiting may mean finding ways around systems that block pathways to care to remain true to the principle of a universal service.

Being prepared to wait requires tolerating uncertainty. This is the suspended time of care: of staying with a situation that is not developing, persisting in the face of chronic presentations, and being prepared to repeat, return, adjust, and endure. When the NHS was conceived, no-one knew how the post-war experiment in democratising the UK’s need for health care would turn out. But perhaps the most important aspect was the care involved in the collectivisation itself, in which everyone became a patient of the NHS, if only “in waiting”. As the NHS faces calls for crisis-driven reform and restructuring, perhaps now is the time for both decision makers and publics to undertake a more nuanced conversation about waiting and care. What waiting can be tolerated, with care? When and how does being made to wait become neglect? How can the service be resourced to undertake care that feels and indeed is timely, including the care that takes place while waiting? And perhaps, most importantly, what principles and practices are worth waiting for?

## References

[R1] Baraitser L (2017). Enduring time.

[R2] Bivins R (2020). Serving the nation, serving the people: echoes of war in the early NHS. Med Humanit.

[R3] Cregeen S, Hughes C, Midgley N, Rhode M, Rustin M, Catty J (2017). Short-term psychoanalytic psychotherapy for adolescents with depression: a treatment manual.

[R4] Gregory A (2023). Child referrals for mental healthcare in England up 39% in a year.

[R5] Hage G (2009). Waiting.

[R6] Kapadia D, Zhang J, Salway S (2022). NHS Race and Health Observatory.

[R7] Janeja KM, Bandak A (2018). Ethnographies of waiting: doubt, hope and uncertainty.

[R8] Koselleck R, Richter MW (2006). Crisis. J Hist Ideas.

[R9] NHS England (2023). Statistical press notice: NHS Referral to Treatment (RTT) waiting times data November 2022.

[R10] Roitman J (2013). Anti-Crisis.

[R11] Royal College of Emergency Medicine (2022). Emergency care in crisis as more patients than ever before face dangerously long waits in Emergency Departments.

[R12] Sheard S (2018). Space, place and (waiting) time: reflections on health policy and politics. Health Econ Policy Law.

[R13] Schwartz B (1975). Queuing and waiting: studies in the social organization of access and delay.

[R14] Waddell M (2018). On adolescence: inside stories.

